# Efficacy of PD-1 or PD-L1 inhibitors for the therapy of cervical cancer with varying PD-L1 expression levels: a single-arm meta-analysis

**DOI:** 10.3389/fonc.2024.1454372

**Published:** 2024-08-20

**Authors:** Jie Yang, Haizan Yu, Yilei Zhang, Mingli Zhu, Mengyu Zhang, Qiming Wang

**Affiliations:** Department of Gynaecology, III, Women’s and Children’s Hospital of Ningbo University, Ningbo, Zhejiang, China

**Keywords:** PD-1inhibitors, PD-L1 expression, cervical cancer, PD-L1 inhibitors, meta-analysis

## Abstract

**Objective:**

To assess the effectiveness and tolerability of both PD-1 and PD-L1 inhibitors in advanced cervical cancer (CC), focusing on varying PD-L1 levels.

**Methods:**

A comprehensive exploration was carried out on EMBASE, PubMed, Cochrane Library databases as well as Web of Science up to May 25, 2024, for studies involving advanced CC patients receiving PD-1/PD-L1 inhibitors. Inclusion criteria were studies reporting objective response rate (ORR), disease control rate (DCR), median progression-free survival (PFS), as well as median overall survival (OS). Data extraction and quality assessment were performed by two reviewers using the JBI Case Series Critical Appraisal Checklist, followed by a meta-analysis via STATA/MP 16.0.

**Results:**

Five eligible studies comprising 223 patients were chosen. ORR and DCR were 42% (95% CI: 17%-66%, P = 0.00) and 70% (95% CI: 22%-117%, P = 0.00), respectively, in the PD-L1 positive patients and were 36% (95% CI: 17%-54%, P = 0.00) and 47% (95% CI: 30%-63%, P = 0.00), respectively, in patients with PD-L1 negativity. For patients exhibiting PD-L1 positivity, median PFS and median OS were 3.98 months (95% CI: 0.80–7.16, P = 0.01) and 11.26 months (95% CI: 3.01–12.58, P = 0.00), respectively.

**Conclusion:**

With PD-1/PD-L1 inhibitors, PD-L1 positive CC patients demonstrate superior ORR, DCR, median PFS, and median OS, underscoring PD-L1 as one biomarker for immunotherapy response.

## Introduction

Cervical cancer (CC) is still a significant contributor to cancer-related mortality in women worldwide, particularly in middle- and low-income countries (1). According to 2020 data, there were approximately 604,127 new cases of cervical cancer worldwide, and 341,831 deaths, with age-standardised incidence and mortality rates of 13.3 and 7.2 per 100,000 women, respectively (2). Despite great progress in both screening and vaccination, a majority of patients still experience serious disease or recurrence and have limited therapy options and unfavourable prognoses (3, 4). Traditional therapies, including chemotherapy, radiation as well as surgery, have presented limited efficacy in these stages of the disease, entailing the exploration of innovative therapy (5).

With the advent of immunotherapy, cancer treatment has been revolutionized bringing hope for patients suffering from advanced tumours. Programmed cell death protein 1 (PD-1) and programmed death-ligand 1 (PD-L1) inhibitors have presented encouraging results in cancers as one class of immune checkpoint inhibitors, including melanoma, bladder cancer as well as non-small cell lung cancer (6, 7). These inhibitors lift the immune system’s capability of recognizing and eliminating cancer cells by disrupting the binding between PD-1 on T cells and PD-L1 on tumour cells (7). The PD-L1 quantification on tumour cells is commonly assessed using the Combined Positive Score (CPS). It has emerged as one potential biomarker for forecasting the reaction to PD-1/PD-L1 inhibitors (8). CPS is determined by assessing the proportion of PD-L1-positive tumour cells and immune ones relative to the total viable tumour ones (9). Preliminary clinical studies indicate a possibility of exhibiting better reactions to PD-1/PD-L1 inhibitors in patients having higher CPS, which implies a potential stratified therapy (10, 11).

The meta-analysis is to assess the effectiveness and tolerability of both PD-1 and PD-L1 inhibitors in treating advanced CC systematically, with a particular focus on different PD-L1 expressions. Data were integrated from various high-quality studies to comprehensively understand the potential of these immune therapies in improving the outcomes of advanced CC patients.

## Methods

Based on implementation under the recommendations of the Cochrane Handbook for Systematic Reviews of Interventions, this study was reported in accordance with the Preferred Reporting Items for Systematic Reviews and Meta-Analyses (12). The current study was formally registered on the International Platform of Registered Systematic Review and Meta-analysis Protocols (INPLASY) (ID: INPLASY202460062).

### Search strategy

We performed an extensive search across various databases like Web of Science, PubMed, EMBASE, as well as the Cochrane Library, encompassing articles published before May 25, 2024. The search was restricted to studies published exclusively in the English language with the following terms for search: “Uterine Cervical Neoplasms” OR “CC” AND “Immune Checkpoint Inhibitors” OR “PD-1 Inhibitor” OR “PD-L1 Inhibitor”. We performed a manual review to the reference lists of the encompassed articles for identifying additional related research. The particular search process is detailed in **Supplementary File 1**.

### Inclusion and exclusion criteria

#### Studies were encompassed if they met the criteria below:

1. Patients were confirmed with advanced or recurrent CC, regardless of subtype.
2. Patients received treatment by PD-1 or PD-L1 inhibitors alone or in conjunction with other therapies.
3. Retrospective analyses or stage II clinical trials.
4. Included studies assessed relevant clinical outcomes, such as PFS, ORR, OS, DCR, as well as AEs, using RECIST 1.1 criteria (13).
5. Tumour PD-L1 was assessed and quantified as one CPS, which was calculated as the percentage of PD-L1-stained cells divided by the sum of viable tumour cells multiplied by 100. The definition of positivity was established as having a CPS of 1 or higher.

#### The exclusion criteria were:

1. Animal research, meta-analyses, reviews, duplicate reports, letters or case reports.
2. Studies with fewer than 10 patients.

Two reviewers conducted a thorough screening of articles independently, assessing their eligibility according to pre-established criteria Disagreements/discrepan were resolved through consensus between the two reviewers or with the assessment of one-third reviewers if necessary.

### Data extraction and quality evaluation

Through one predefined extraction form, two reviewers extracted data. The extracted data encompassed baseline patient characteristics, study characteristics, and predefined outcomes (ORR, DCR, PFS, OS). The quality of clinical studies was evaluated via the JBI Case Series Critical Appraisal Checklist (14).

### Statistical analyses

Analyses were conducted via STATA/MP 16.0. Inter-study heterogeneity was judged via the chi-square test as well as the I² statistic. Random-effects models (REM) were adopted when I²≥50% (indicating high heterogeneity), and fixed-effects models (FEM) were adopted when I²<50% (implying low heterogeneity) (15). The robustness of the pooled results was judged via sensitivity analyses. Egger’s test was conduc to evaluate the possible publication bias.

## Results

### Literature search

**Figure 1:**
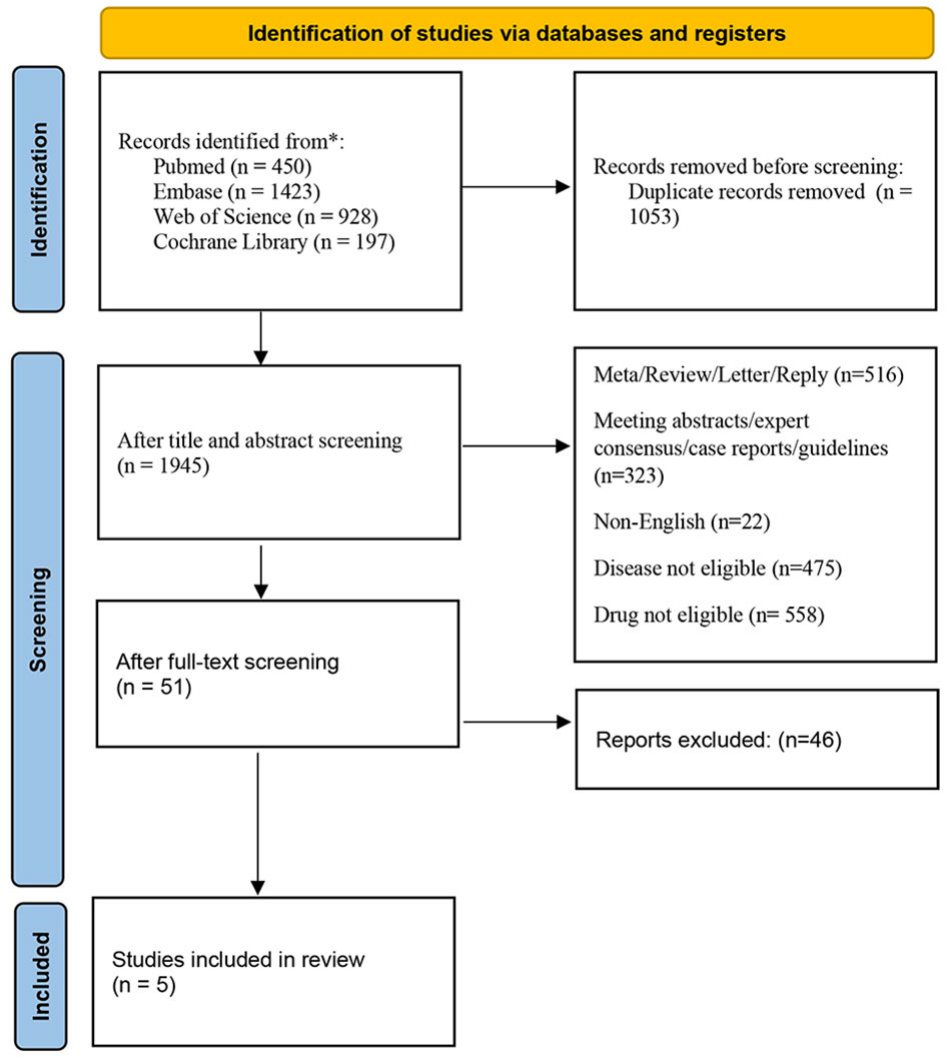
The flow diagram of studies is included in this meta-analysis.

The initial search strategy yielded 2,998 relevant articles. After removing 1,053 duplicate studies, we screened titles and abstracts, causing the exclusion of 1,894 studies not fulfilling the inclusion criteria. Subsequently, we performed a detailed review of the whole texts of the left 51 potentially eligible papers, and ultimately selected 5 trials for the final analysis (16–19). The process of selecting studies is depicted in **Figure 1**. All eligible research data were obtained from published manuscripts.

### Study characteristics

Totally, 5 studies were included in the final analysis **Table 1** presents their detailed characteristics.

**Table 1:** Characteristics of studies included in this meta-analysis.

Study	Year	Study type	Stage	Age	Intervention types	Number of patients	PD-L1 CPS≥1%	PD-L1 CPS<1%	PD-L1 CPS unknown	Follow-up (m), median (range)
Chunyan Lan	2024	NRCT single-arm, phase II	metastatic, recurrent, or persistent cervical cancer	51 (33–67)	Camrelizumab	45	10	30	5	6 (0.97–37.4)
Yin Wang	2023	NRCT single-arm, phase II	recurrent or metastatic cervical cancer	50 (34–68)	Sintilimab	27	18	5	4	10.2 (3.0–24.5)
Lingfang Xia	2022	NRCT single-arm, phase II	recurrent or metastatic cervical cancer	50 (43–55)	Camrelizumab	33	10	9	14	13.6 (10.0–23.6)
Hyun Cheol Chung	2019	international, open-label, multicohort	advanced Cervical Cancer	46 (24–75)	Pembrolizumab	98	82	15	1	10.2 (0.6–22.7)
Kenji Tamura	2019	prospective, multicenter, open-label	advanced or recurrent uterine cervical cancer	50 (32–68)	Nivolumab	20	5	15	/	5.4 (1.0–13.9)

### Quality assessment

On the basis of the JBI Critical Appraisal Checklist for Case Series, five clinical studies were evaluated, comprising ten items that examine the quality of case reports including case selection, evaluation of the disease or health problem, and case data presentation. The assessment results are provided in **Table 2**.

**Table 2:** The JBI Critical Appraisal Checklist for Case Series.

Query	Chunyan Lan	Yin Wang	Lingfang Xia	Hyun Cheol Chung	Kenji Tamura
Were there clear criteria for inclusion in the case series?	YES	YES	YES	YES	YES
Was the condition measured in a standard, reliable way for all participants included in the case series?	YES	YES	YES	YES	YES
Were valid methods used for the identification of the condition for all participants included in the case series?	YES	YES	YES	YES	YES
Did the case series have consecutive inclusion of participants?	UNCLEAR	YES	YES	YES	UNCLEAR
Did the case series have a complete inclusion of participants?	YES	YES	YES	YES	YES
Was there clear reporting of the demographics of the participants in the study?	YES	YES	YES	YES	YES
Was there clear reporting of clinical information of the participants?	YES	YES	YES	YES	YES
Were the outcomes or follow-up results of cases clearly reported?	YES	YES	YES	YES	YES
Was there clear reporting of the presenting site(s)/clinic(s) demographic information?	YES	YES	YES	YES	YES
Was statistical analysis appropriate?	YES	YES	YES	YES	YES
Overall appraisal	Include	Include	Include	Include	Include

### Meta-analysis results

#### Comparison of ORR by PD-L1 CPS

**Figure 2:**
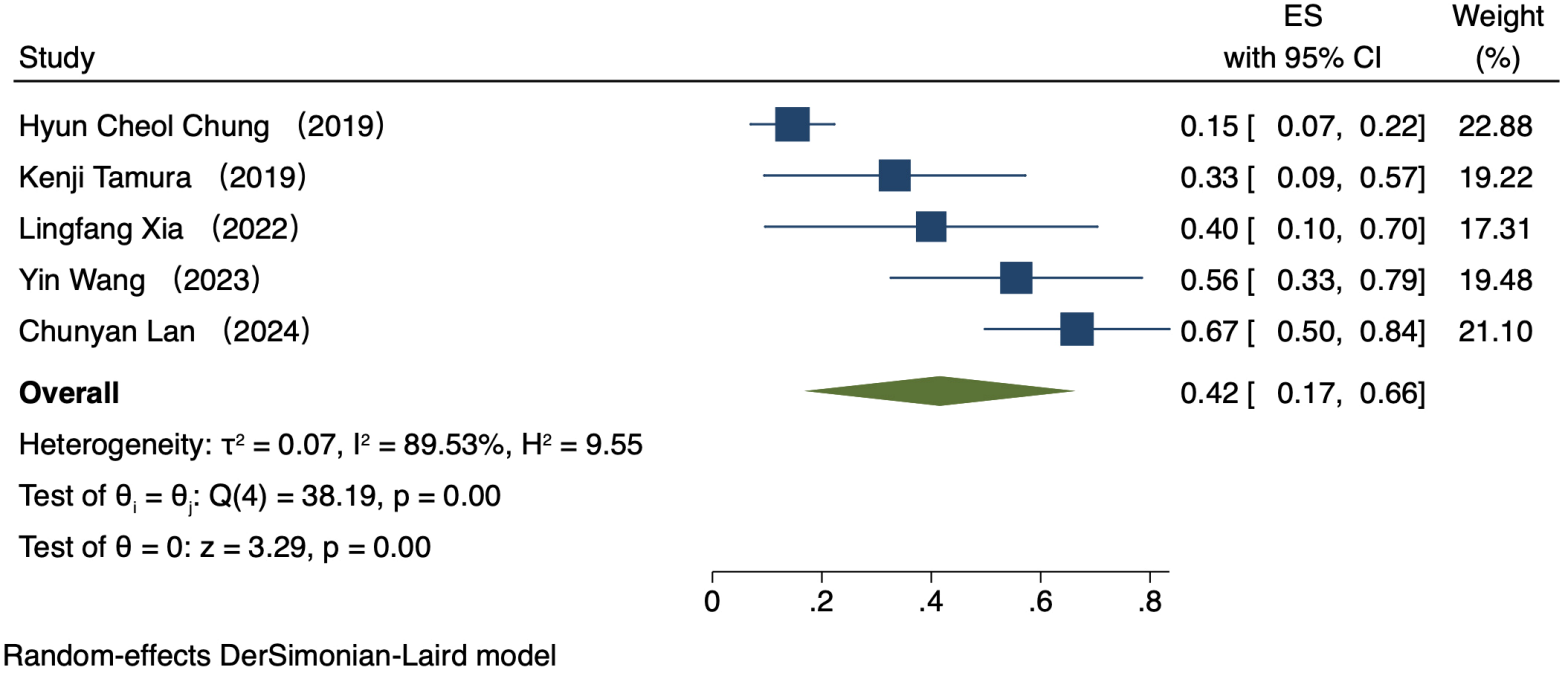
Forest plot of ORR in PD-L1 positive.

**Figure 3:**
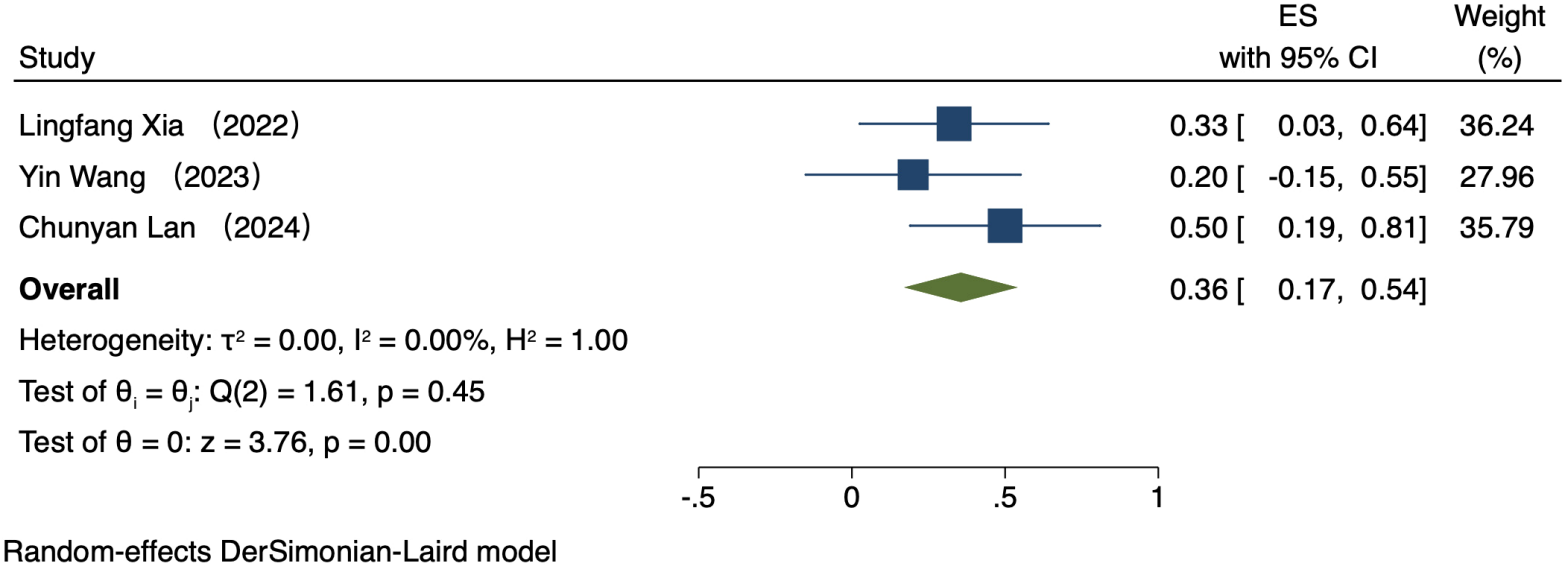
Forest plot of ORR in PD-L1 negative.

Five studies (223 patients) analyzed ORR by PD-L1 CPS (16–20). In patients exhibiting PD-L1 positivity, a REM was used because of notable heterogeneity (I² = 89.53%, P = 0.00). The ORR was 42% (95% CI: 17%-66%, P = 0.00, **Figure 2**). In patients exhibiting PD-L1 negativity, a FEM was used because of low heterogeneity (I² = 0.00%, P = 0.45). The ORR was 36% (95% CI: 17%-54%, P = 0.00, **Figure 3**).

#### Comparison of DCR by PD-L1 CPS

**Figure 4:**
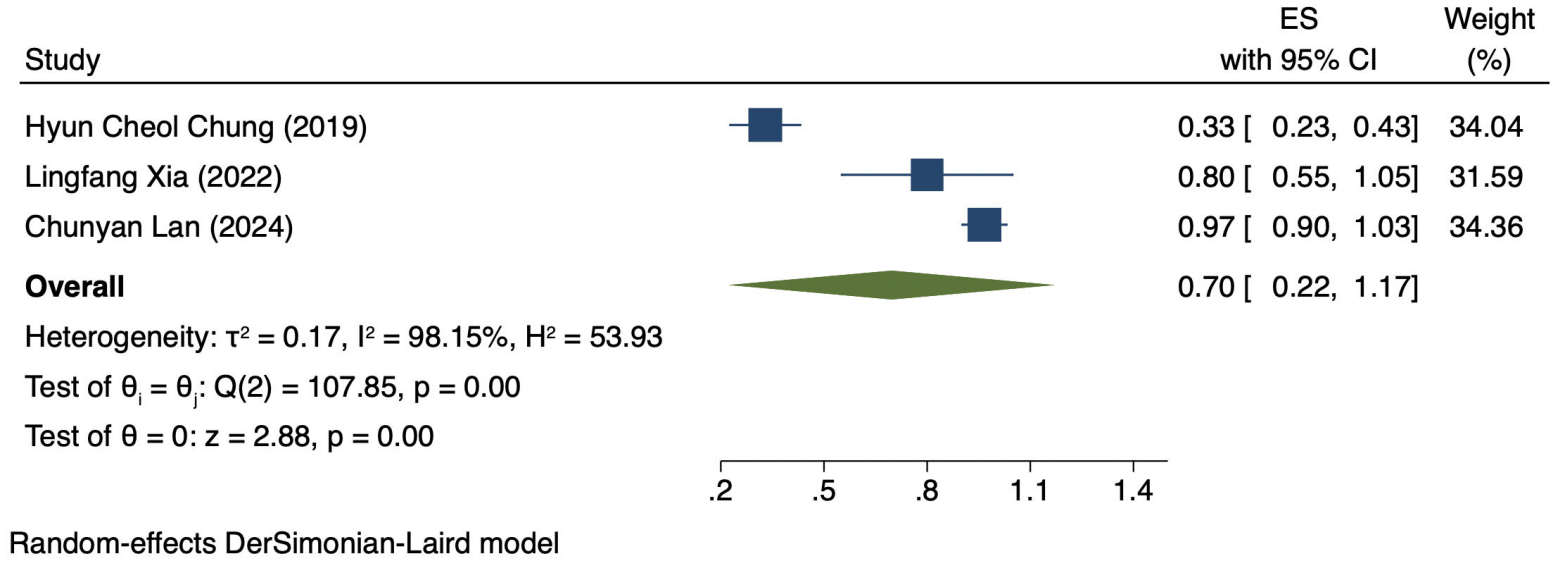
Forest plot of DCR in PD-L1 positive.

**Figure 5:**
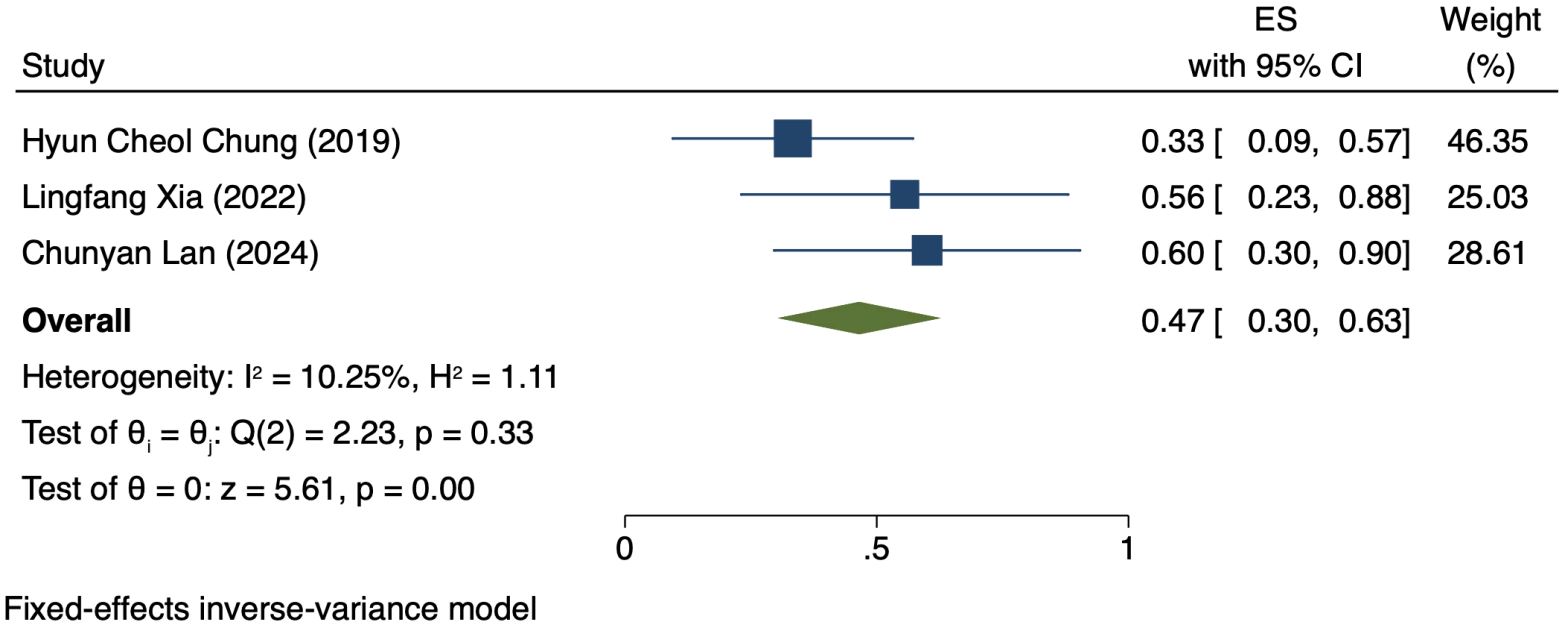
Forest plot of DCR in PD-L1 negative.

Three studies (176 patients) analyzed DCR by PD-L1 CPS (17, 19, 21). In PD-L1 positive patients, a REM was used because of notable heterogeneity (I² = 98.15%, P = 0.00). The DCR was 70% (95% CI: 22%-117%, P = 0.00), as shown in **Figure 4**. In PD-L1 negative patients, a FEM was used because of low heterogeneity (I² = 10.25%, P = 0.33). The DCR was 47% (95% CI: 30%-63%, P = 0.00), as shown in **Figure 5**.

### Median PFS in patients exhibiting PD-L1 positivity

**Figure 6:**
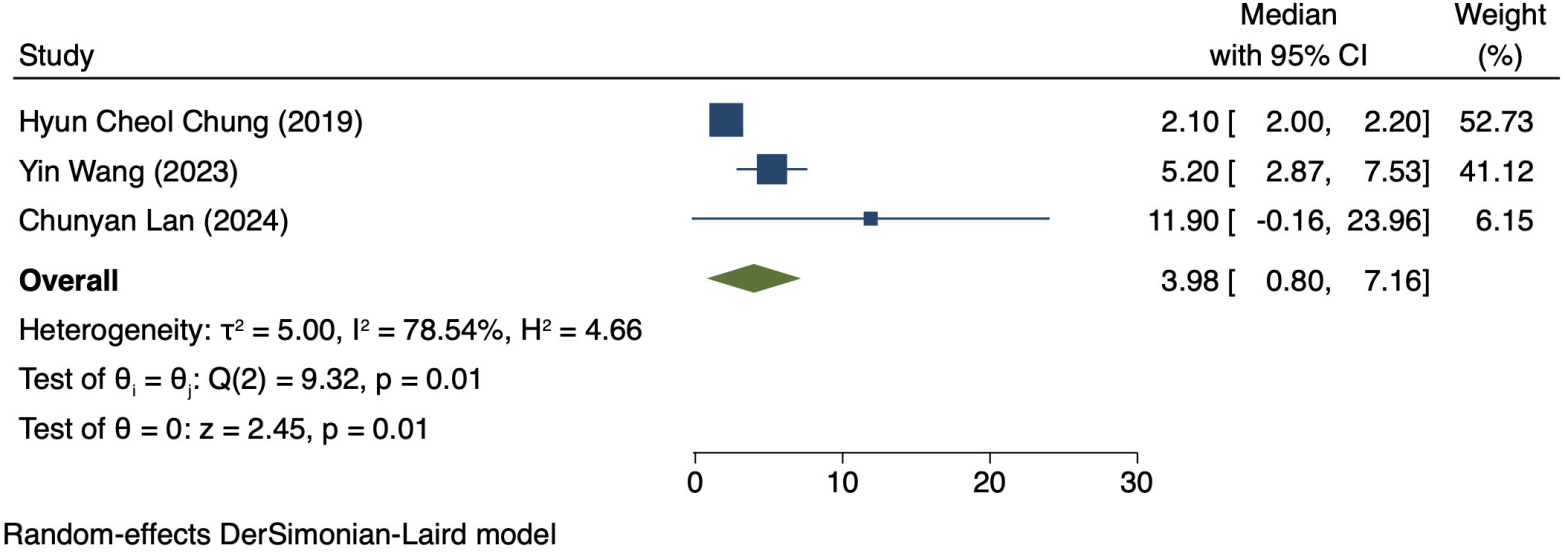
Forest plot of PFS in PD-L1 positive.

Three studies (170 patients) analyzed PFS in Patients exhibiting PD-L1 positivity (16, 17, 20). A REM was used because of notable heterogeneity (I² = 78.54%, P = 0.01). The PFS was 3.98 months (95% CI: 0.80–7.16, P = 0.01), as shown in **Figure 6**.

### Median OS in patients exhibiting PD-L1 positivity

**Figure 7:**
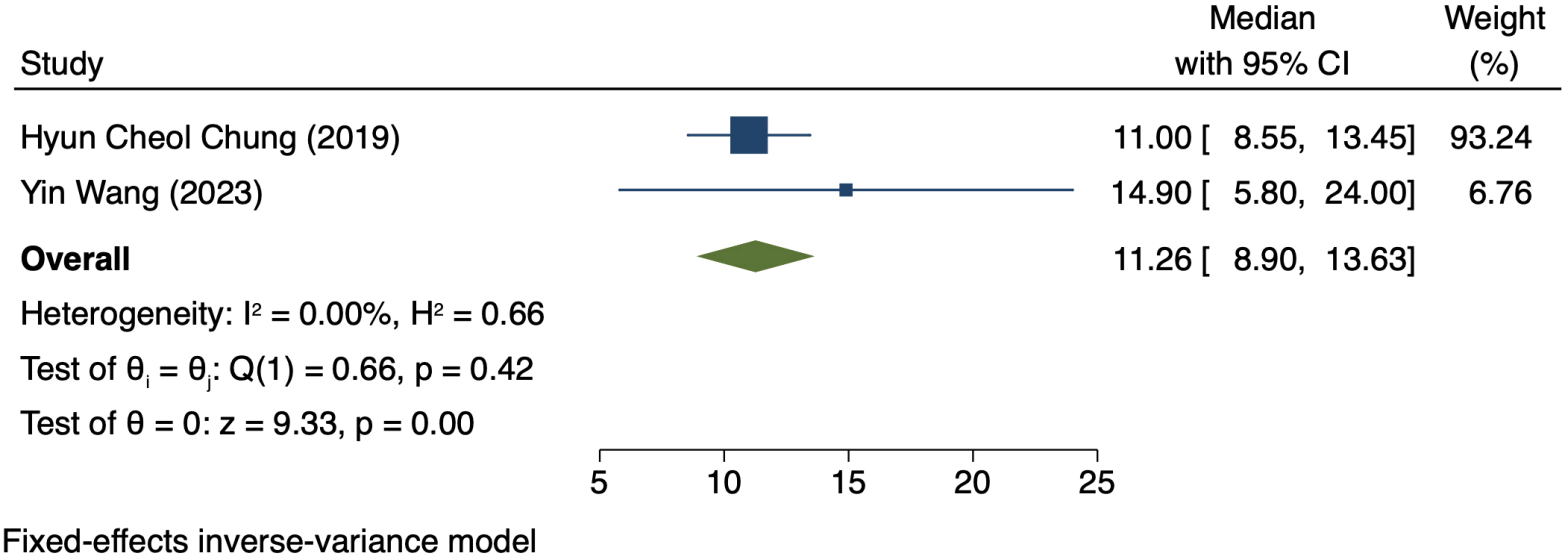
Forest plot of OS in PD-L1 positive.

Two studies (125 patients) analyzed OS in patients exhibiting PD-L1 positivity (16, 20). A FEM was used due to low heterogeneity (I² = 0.00%, P = 0.42). The OS was 11.26 months (95% CI: 3.01–12.58, P = 0.00, **Figure 7**).

### Sensitivity analysis

By sequentially excluding each study, a sensitivity analysis was performed for assessing its impact on the summary results. According to the analysis results, no individual study significantly impacts the overall 95% CI of the summary results, indicating a relatively robust of the meta-analysis results. The results are presented in **Supplementary File 2**.

### Publication bias

To ensure the validity of the meta-analysis, publication bias was judged via Egger’s test. The p-value of 0.79 (> 0.05), indicates no notable publication bias.

## Discussion

This study comparatively analyzed ORR and DCR among patients who had different PD-L1 CPS, focusing on assessing the efficacy disparity between groups exhibiting PD-L1 positivity and PD-L1 negativity. The results revealed an ORR of 42% (95% CI: 17%-66%) and 36% (95% CI: 17%-54%) in the group exhibiting PD-L1 positivity and group exhibiting PD-L1 negativity, respectively. This difference suggests a possibly larger response rate of PD-L1-positive patients to immunotherapy. The underlying mechanism for it can be explained by the interaction between PD-L1 with the immune system. PD-L1, a cell surface protein frequently found on tumour cells, binds to the PD-1 receptor on T cells, suppressing the activity of T cells and helping tumour cells evade immune system attacks (22, 23). In tumours expressing PD-L1, tumour cells can more effectively utilize this mechanism to evade immune surveillance. Thus, these patients possibly have a better response to immune checkpoint inhibitors like PD-1/PD-L1 inhibitors, as these drugs are able to disrupt the binding of PD-1/PD-L1 with restore T cell-mediated tumour attack (24, 25). DCR was also compared among patients who had different PD-L1 CPS. The group exhibiting PD-L1 positivity and group exhibiting PD-L1 negativity had a DCR of 70% (95% CI: 22%-117%) and 47% (95% CI: 30%-63%), respectively. These findings imply the high value of PD-L1 expression in immune therapy response further (26). for more deeply probing into the survival outcomes of patients exhibiting PD-L1 CPS positivity, we analyzed the PFS and OS and found a PFS and OS of 3.98 months (95% CI: 0.80–7.16) and 7.80 months (95% CI: 3.01–12.58), respectively, in patients exhibiting PD-L1 CPS positivity. The findings imply the possibility of experiencing improved long-term survival rates among PD-L1 CPS-positive patients receiving immune therapy (27–29).

These results underscore the high value of PD-L1 in immune therapy. Patients exhibiting PD-L1 positivity demonstrated better efficacy in multiple key outcome measures in contrast to patients exhibiting PD-L1 negativity, indicating PD-L1 as an effective biomarker for identifying patients with a larger likelihood of favorable response to immune therapy in patients (30, 31).

Whereas, the current research also has certain limitations. First, a noticeable heterogeneity in the analysis could affect the stability of the results. Second, the included studies with relatively small sample sizes mostly consisted of non-controlled trials, limiting the generalizability and persuasiveness of the findings. Additionally, because of lack of enough pathological data, we could not further investigate the treatment response based on different types of CC tissue. studies included in this analysis predominantly involved Asian patients, raising uncertainty about the generalizability of these findings to other populations. Therefore, further validation of these findings is warranted through the implementation of large-scale randomized controlled trials (RCTs) in the future (32, 33).

In conclusion, PD-L1 expression is crucial in immune therapy, with PD-L1 CPS-positive patients demonstrating better efficacy in terms of ORR, DCR, median PFS, and median OS in contrast to patients exhibiting PD-L1 negativity. While the initial findings are encouraging, additional research is required to ascertain the wide applicability as well as long-term implications of these findings (34).

## Conclusion

The meta-analysis verifies that CC patients exhibiting PD-L1 positivity have superior efficacy regarding ORR, DCR, median PFS, as well as median OS when receiving PD-1/PD-L1 inhibitor therapy in contrast to patients exhibiting PD-L1 negativity. These findings support the utilization of PD-L1 as one biomarker for forecasting the advanced CC patients’ reaction to immunotherapy.

## Data availability statement

The original contributions presented in the study are included in the article/**Supplementary Material**. Further inquiries can be directed to the corresponding author.

## Author contributions

JY: Conceptualization, Data curation, Investigation, Writing – original draft. HY: Investigation, Methodology, Writing – original draft. YZ: Data curation, Formal analysis, Writing – original draft. MlZ: Formal analysis, Investigation, Writing – original draft. MyZ: Formal analysis, Investigation, Project administration, Writing – original draft. QW: Conceptualization, Supervision, Writing – review & editing.

## Supplementary material

Supplementary File 1 Search strategy.

Supplementary File 2 Sensitivity Analysis.
