# Supplementary figures and images for: Efficacy of PD-1 or PD-L1 inhibitors for the therapy of cervical cancer with varying PD-L1 expression levels: a single-arm meta-analysis
Source: Front Oncol. 2024 Aug 20;14:1454372. doi: 10.3389/fonc.2024.1454372 (PMC11368785; doi:10.3389/fonc.2024.1454372)

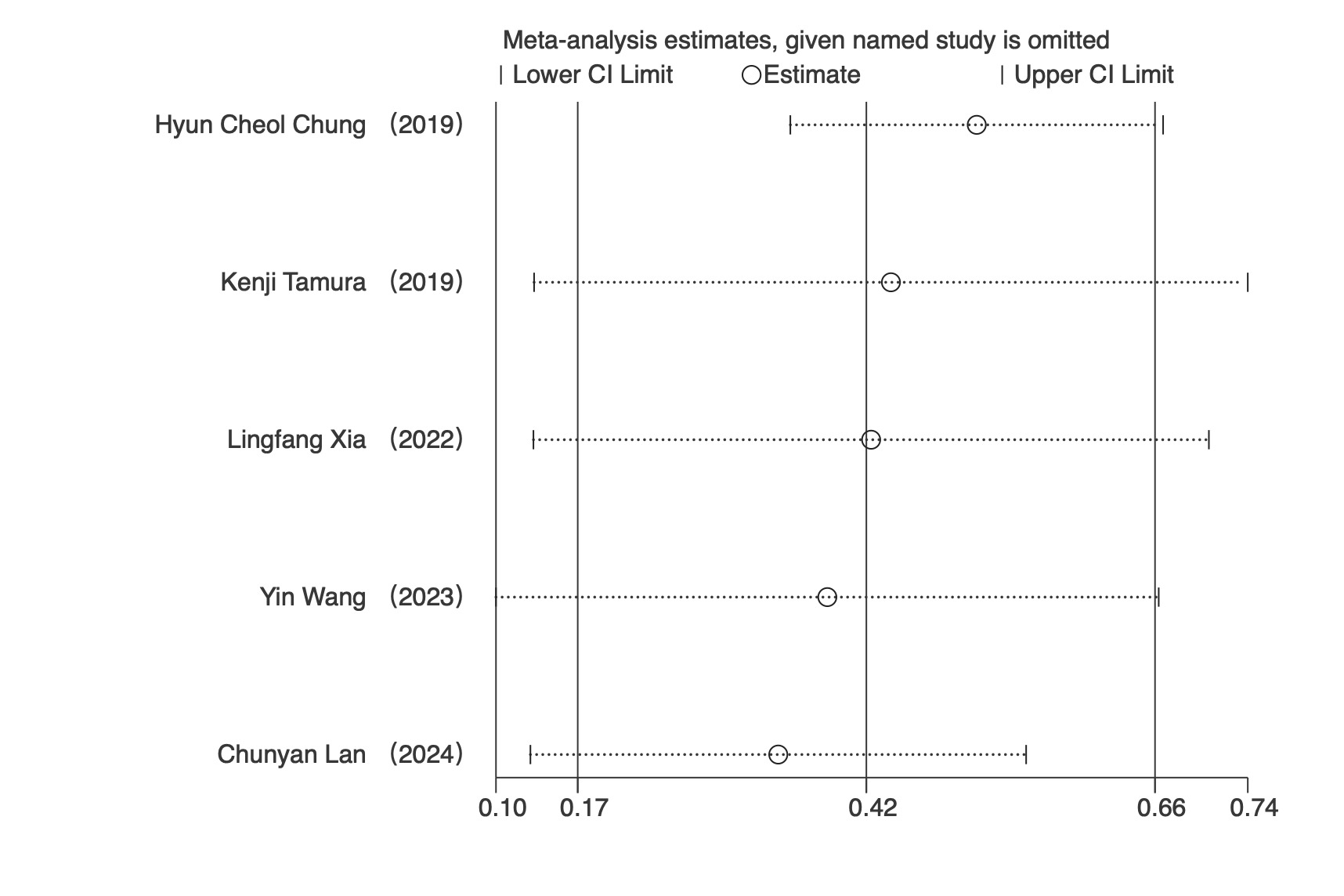

Supplement: Supplementary File 1 — Search strategy. [file Image1.jpeg]

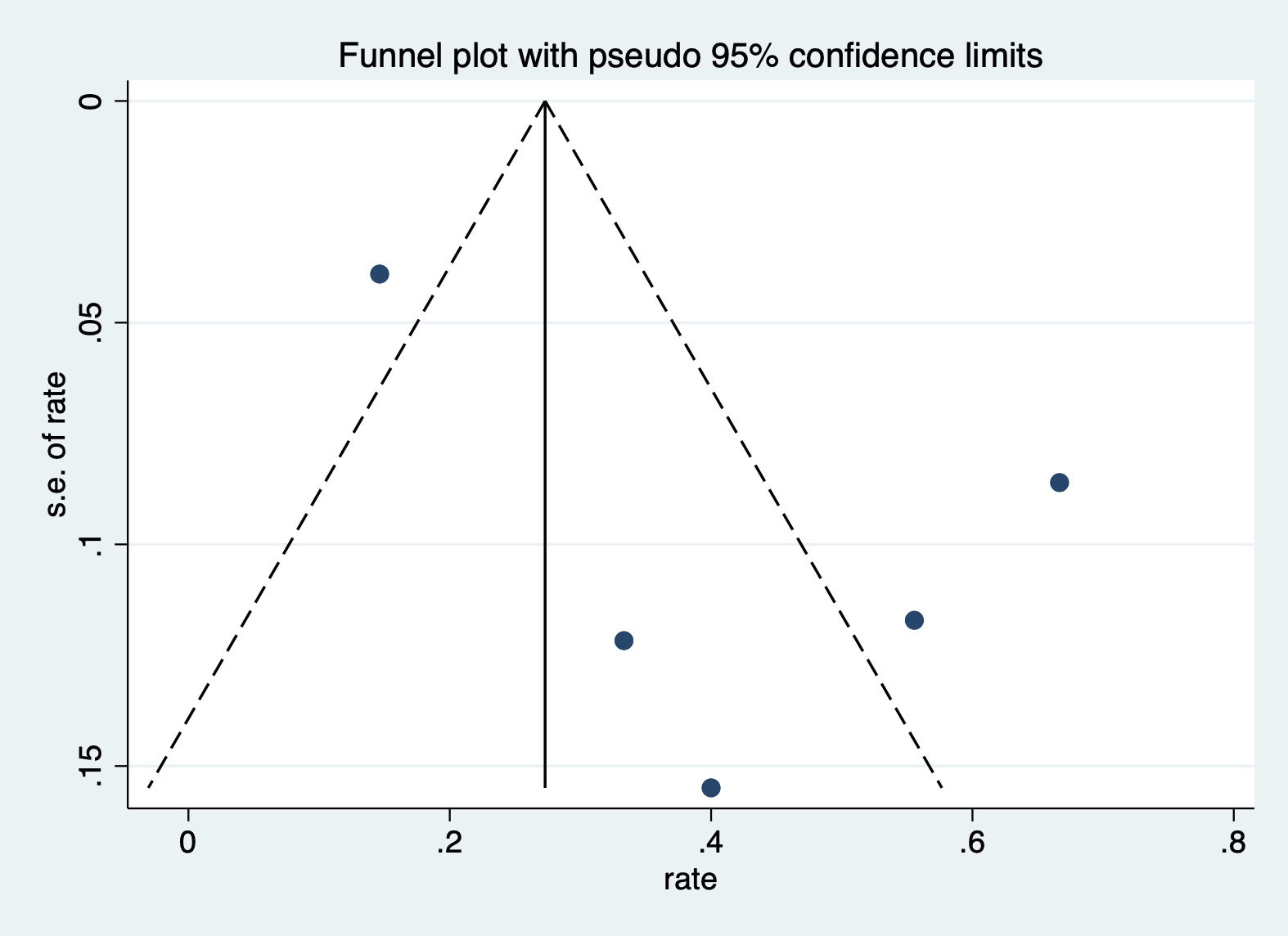

Supplement: Supplementary File 2 — Sensitivity Analysis. [file Image2.jpeg]
